# Complete Genome Sequence of *Endomicrobium proavitum*, a Free-Living Relative of the Intracellular Symbionts of Termite Gut Flagellates (Phylum *Elusimicrobia*)

**DOI:** 10.1128/genomeA.00679-15

**Published:** 2015-07-16

**Authors:** Hao Zheng, Andreas Brune

**Affiliations:** a Department of Biogeochemistry, Max Planck Institute for Terrestrial Microbiology, Marburg, Germany; b LOEWE Center for Synthetic Microbiology, SYNMIKRO, Philipps-Universität Marburg, Marburg, Germany

## Abstract

We sequenced the complete genome of *Endomicrobium proavitum* strain Rsa215, the first isolate of the class *Endomicrobia* (phylum *Elusimicrobia*). It is the closest free-living relative of the endosymbionts of termite gut flagellates and thereby provides an excellent model for studying the evolutionary processes during the establishment of an intracellular symbiosis.

## GENOME ANNOUNCEMENT

Endomicrobia (formerly termite group 1) form a deep-branching clade of uncultivated bacteria in the *Elusimicrobia* phylum (1, 2), which is so far represented by only a single isolate, *Elusimicrobium minutum* (3). Members of this clade have been identified as intracellular symbionts of termite gut flagellates and are specific for and vertically transferred by their respective hosts (4–6). However, the recovery of 16S rRNA genes of endomicrobia from artificially defaunated or flagellate-free termites and cockroaches indicated the existence of putatively free-living relatives in the same habitat (7, 8). We isolated the strictly anaerobic ultramicrobacterium *Endomicrobium proavitum* strain Rsa215, the first representative of the class *Endomicrobia*, from a sterile-filtered gut homogenate of *Reticulitermes santonensis* (9).

The genome sequence of *E. proavitum* was obtained with Pacific Biosciences (PacBio) single-molecule real-time (SMRT) sequencing. Genomic DNA of strain Rsa215 was prepared using cetyltrimethylammonium bromide (CTAB) extraction (10) and commercially sequenced (GATC Biotech AG, Germany) on a PacBio RS platform using three SMRT cells (insert size, 8 to 12 kbp). Reads were assembled using the PacBio SMRT Portal software (version 2.1.0) and the HGAP assembly algorithm (11). The initial assembly yielded seven contigs with >20× average coverage. Reassembly with Minimus 2 (12) identified overlaps of all contigs, which were confirmed by PCR and Sanger sequencing experiments. The final assembly resulted in a circular chromosome (1,588,979 bp, 39% G+C content) and was confirmed using the latest version of the assembler (HGAP 3). Plasmids were not detected.

Annotation on the MicroScope platform (13) resulted in 1,341 predicted protein-coding genes, 46 tRNA genes, and a single set (16S, 23S, and 5S) of rRNA genes. Many genes of *E. proavitum* were highly similar to those of the closely related “*Candidatus* Endomicrobium trichonymphae” strain Rs-D17 (14), which is an endosymbiont of the large flagellates that cooccur in the same habitat (4). Although the two organisms share many metabolic pathways, the genome of the endosymbiont is much smaller (1,125,857 bp) and highly degraded, and numerous pathways (e.g., those for initiation and regulation of chromosomal replication, lipopolysaccharide biosynthesis, ammonium transport, and assimilation) are interrupted by pseudogenes (14). The same pathways are intact in the free-living *E. proavitum*, which shows no obvious signs of genome reduction but possesses additional functions absent in the endosymbiont, including a set of genes required for nitrogen fixation (*nifHDK*). Comparative genome analysis of the two strains will provide a better understanding of the evolutionary processes that started when their common free-living ancestor became associated with its flagellate host.

### Nucleotide sequence accession number. 

The sequence data for the genome have been deposited in DDBJ/EMBL/GenBank under the accession no. CP009498.
